# Fear of Falling and Its Association with Lower Limb Strength and Balance Control in Elderly Day-Care Center Users

**DOI:** 10.3390/bioengineering13101180

**Published:** 2026-10-09

**Authors:** Jong-Seon Oh, Seong-Gil Kim

**Affiliations:** Department of Physical Therapy, College of Health and Life Science, Korea National University of Transportation, Jeungpyeong-gun 27909, Republic of Korea; dhwhdtjs03335@gmail.com

**Keywords:** fear of falling, elderly day-care center users, lower limb strength, balance control, postural stability, mobility limitation, fall risk assessment, rehabilitation, preventive strategies

## Abstract

This cross-sectional study examined associations between lower limb strength, mobility, balance control, and fear of falling (FOF) among older adults attending community-based day-care centers. Seventy-three participants aged ≥65 years underwent assessments of knee extension strength (KES), Timed Up and Go (TUG), Functional Reach Test (FRT), and the Korean Falls Efficacy Scale-International (FES-I). Pearson correlation and simple linear regression analyses were performed. KES was negatively correlated with FES-I (r = −0.421, *p* < 0.01), TUG was positively correlated with FES-I (r = 0.394, *p* < 0.01), and FRT was negatively correlated with FES-I (r = −0.361, *p* < 0.01). Separate simple linear regression analyses likewise showed significant unadjusted associations of FES-I with KES, TUG, and FRT (all *p* < 0.01). These findings indicate that lower limb strength, mobility, and balance control are significantly associated with FOF. The results support the potential relevance of jointly assessing strength, mobility, balance control, and FOF in older adults attending day-care centers.

## 1. Introduction

The rapid progression of population aging has brought falls to the forefront as one of the most critical health issues among older adults. A fall is not merely an accidental event but a multifactorial problem that contributes to physical decline, psychological distress, and increased social dependency in this population [1]. Falls are a leading cause of injury-related hospitalization among older adults, and recurrent falls can further lead to greater functional dependence and reduced quality of life. Therefore, fall prevention requires a comprehensive approach that encompasses both physical rehabilitation and psychological support.

Age-related declines in lower limb function reflect changes in both muscle mass and muscle quality. Longitudinal evidence indicates that knee extensor strength declines more rapidly than leg lean mass in older adults, suggesting that loss of muscle quantity alone does not fully explain declining strength [2]. Reduced physical activity may further impair muscle maintenance: experimentally reducing daily steps for two weeks decreased leg lean mass and the postprandial muscle protein synthetic response in healthy older adults [3]. Cognitive processes, including attention, executive function, and memory, are also associated with gait performance [4]. Furthermore, a longitudinal study of Korean older adults found that coexisting declines in grip strength and cognitive function were associated with fear of falling (FOF) and related activity restriction [5]. These findings provide a physiological and functional context for examining the relationships between physical performance and FOF.

FOF is a prevalent psychological concern among older adults and may occur even in the absence of a previous fall event [6]. Individuals with elevated FOF tend to restrict their daily physical activities to avoid falling, which may contribute to further muscle weakness and balance deterioration, thereby increasing fall risk. This process creates a vicious cycle characterized by “activity restriction, muscle weakness, balance impairment, and increased fall risk” [7,8]. FOF has also been associated with poorer health-related quality of life and depressive symptoms [9,10]. Thus, these observations support considering both physical function and FOF in clinical assessment.

Community-based day-care centers offer a setting in which physical performance and concerns about falling can be considered together in older adults receiving daytime care. Previous research has shown that perceived and physiological fall risk do not always correspond in older adults [11]. This suggests that functional test performance alone may not fully capture an individual’s concerns about falling. Examining the associations between strength, mobility, balance control, and FOF within day-care settings may therefore provide clinically relevant information for interpreting physical and self-reported assessments together.

Therefore, this cross-sectional study aimed to examine the associations of knee extension strength (KES), mobility assessed using the Timed Up and Go (TUG) test, and balance control assessed using the Functional Reach Test (FRT) with FOF in older adults attending community-based day-care centers. Specifically, the study sought to characterize the individual unadjusted associations of these physical measures with FOF and to provide information relevant to their joint assessment in this care setting.

## 2. Materials and Methods

### 2.1. Study Design

This cross-sectional correlational study investigated the relationships between KES, mobility (Timed Up and Go, TUG), balance control, and FOF in older adults attending community-based day-care centers. The study adhered to the STROBE (Strengthening the Reporting of Observational Studies in Epidemiology) guidelines, and all procedures were approved by the Institutional Review Board of Sunmoon University (SM-202409-022-2).

### 2.2. Participants

Participants were older adults aged 65 years or older enrolled in four day-care centers located in the Chungcheong region of South Korea. Inclusion criteria were as follows: a score of ≥20 on the Korean version of the Mini-Mental State Examination (MMSE-K) and ability to walk independently.

Exclusion criteria included a history of lower limb surgery or fracture within the past six months, severe visual or hearing impairment, and difficulty participating in the measurement procedures. A total of 78 individuals were initially recruited; after excluding five participants owing to incomplete data, 73 participants were included in the final analysis. All participants received a detailed explanation of the study and provided written informed consent.

### 2.3. Measurements and Procedures

All measurements were conducted in the physical therapy rooms of the participating day-care centers under standardized environmental conditions. Two trained physical therapists performed the assessments following identical procedures. The measurement sequence was as follows: baseline information, muscle strength, balance, mobility, and FES-I questionnaire.

A rest period of 1–2 min was provided between assessments.

#### 2.3.1. Knee Extension Strength

Knee extension strength (KES) was assessed using a hand-held dynamometer (MicroFET2; Hoggan Scientific LLC, Salt Lake City, UT, USA) [12,13]. Participants sat on a chair with their knees flexed at 90°, and the examiner positioned the dynamometer against the anterior aspect of the ankle of the leg being tested. The 90° knee-flexion position standardized the testing position across participants and trials and was consistent with a previously reported seated hand-held dynamometry protocol in older adults [14]. Participants were instructed to perform an isometric knee extension for 5 s. Each leg was tested three times, and the mean of the three trials was calculated separately for each leg. The participant-level KES value used in the analysis was the average of the right- and left-leg mean values and was expressed in kilogram-force (kgf).

#### 2.3.2. Mobility (Timed Up and Go)

Mobility was assessed using the TUG test. Participants were instructed to stand up from a chair with armrests, walk 3 m, turn around, return to the chair, and sit down. The time required to complete the task was recorded in seconds [15]. The test was performed three times, and the mean value was used for analysis. This test is widely used as a standardized assessment of functional mobility and fall risk in older adults.

#### 2.3.3. Balance Control (Functional Reach Test)

Balance control was evaluated using the FRT [16]. Participants stood alongside a wall with a measuring tape affixed horizontally and extended one arm forward at shoulder height. The maximum forward reach distance achieved without losing balance was recorded in centimeters. The highest value among three trials was used for analysis. The FRT serves as an indirect indicator of dynamic balance and fall risk.

#### 2.3.4. Fear of Falling

FOF was assessed using the Korean version of the Falls Efficacy Scale-International (FES-I) [17,18]. The scale consists of 16 items rated on a 4-point Likert scale, yielding a total score ranging from 16 to 64, with higher scores indicating greater FOF.

#### 2.3.5. Fall Experience

Fall experience within the past 12 months was assessed using a dichotomous (yes/no) question. A fall was defined as “an event in which an individual unintentionally comes to rest on the ground or a lower level.”

### 2.4. Statistical Analysis

All statistical analyses were performed using SPSS Statistics 26.0 (IBM Corp., Armonk, NY, USA). The normality of continuous variables was evaluated using the Shapiro–Wilk test, and results were presented as mean ± standard deviation. Categorical variables were presented as frequencies and percentages. Pearson correlation analysis was conducted to examine the relationships among KES, TUG, FRT, and FES-I scores.

Separate simple linear regression analyses were conducted with the FES-I score as the dependent variable and KES, TUG, or FRT as the sole explanatory variable in each model. Regression coefficients (β), t statistics, and *p* values were reported. The coefficient of determination (R^2^) was used to summarize the proportion of variance in FES-I scores explained by each model. All three models were unadjusted. Fall history was summarized descriptively and was not included as a covariate. The analyses characterized individual unadjusted associations. A two-tailed *p* value < 0.05 was considered statistically significant.

## 3. Results

A total of 73 older adults were included in the final analysis (Table 1). The mean age of the participants was 79.2 ± 5.1 years; 17 were male (23.3%) and 56 were female (76.7%). The mean BMI was 23.4 ± 3.2 kg/m^2^. The mean values for the primary outcome measures were as follows: KES, 11.8 ± 4.3 kgf; TUG, 13.6 ± 3.8 s; FRT, 20.9 ± 6.4 cm; and FES-I, 33.5 ± 9.1 points. In total, 25 participants (34.2%) reported experiencing at least one fall within the past 12 months.

Pearson correlation analysis indicated that KES was significantly negatively correlated with FES-I (r = −0.421, *p* < 0.01), suggesting that reduced lower limb strength was associated with greater FOF. TUG demonstrated a significant positive correlation with FES-I (r = 0.394, *p* < 0.01), indicating that longer TUG times were associated with higher levels of FOF. FRT also showed a significant negative correlation with FES-I (r = −0.361, *p* < 0.01). Collectively, these findings suggest that reduced muscle strength, impaired mobility, and poorer balance control are associated with greater FOF (Table 2).

Simple linear regression analyses showed significant unadjusted associations of FES-I with KES (β = −0.421, t = −3.91, *p* < 0.001, R^2^ = 0.177), TUG (β = 0.394, t = 3.61, *p* < 0.001, R^2^ = 0.155), and FRT (β = −0.361, t = −3.26, *p* = 0.002, R^2^ = 0.130) (Table 3).

Overall, these results indicate that lower limb strength, mobility, and balance control each showed significant unadjusted associations with FOF in older adults attending day-care centers.

## 4. Discussion

This cross-sectional study found that lower KES, longer TUG completion times, and shorter FRT distances were associated with higher FES-I scores among older adults attending community-based day-care centers. These findings indicate that knee extensor strength, functional mobility, and balance control during forward reaching are each related to FOF in this sample. The results represent individual unadjusted associations and should not be interpreted as independent effects.

Lower KES was associated with greater FOF, consistent with the lower knee extensor strength observed among new long-term care enrollees who reported FOF [19]. Their broader care population and use of self-reported fear and the original Falls Efficacy Scale limit direct comparison with the present FES-I-based findings. KES assesses isometric knee extensor force, whereas the TUG incorporates rising from a chair, walking, turning, and sitting [15]. A positive association between TUG time and scores on the original Falls Efficacy Scale has also been reported in older Korean women, although the sample and FOF measure differed from those in the present study [20]. Thus, the results relate FOF to both a specific strength measure and functional mobility performance, without establishing whether either measure provides additional information beyond the other.

Shorter FRT distances were associated with greater FOF (r = −0.361). A similar negative correlation between functional reach and FES-I-BRAZIL scores was reported in community-dwelling older adults (r = −0.516) [21]. The direction of association is consistent, although differences in participant characteristics and care settings limit direct comparison of its magnitude. As a forward-reaching test, the FRT provides a clinical estimate of the margin of stability [16]. The present finding therefore relates concerns about falling to balance control during forward reaching. FRT performance and FES-I scores nevertheless represent different information: performance in one standardized task and concerns across daily activities. Their association does not imply that FRT captures all dimensions of balance or FOF.

The relationship between physical performance and FOF may be bidirectional. Physical limitations may increase concerns about falling, while fear-related avoidance may contribute to reduced activity. A theoretical account proposes that FOF-related changes in attention and postural stiffening may compromise performance during complex movement tasks [22]. Previous research has also reported associations between FOF and frailty, and between FOF accompanied by activity restriction and sarcopenia [8]. These findings and theoretical explanations provide a possible context for the present associations. However, these mechanisms and behavioral pathways were not examined in this study, and the temporal direction of the observed relationships cannot be determined.

From a clinical perspective, the findings support the potential relevance of considering KES, TUG, and FRT results alongside FES-I scores in day-care settings. FRT and TUG have also been used to assess balance and mobility in older adults with dementia attending day-care centers [23]. However, physical findings and concerns about falling do not necessarily correspond, as demonstrated by previously reported discrepancies between perceived and physiological fall risk [11]. For example, relatively preserved physical performance may coexist with substantial concerns about particular activities. Considering both findings could prompt discussion of previous falls and situations that cause concern, helping clinicians contextualize the person’s test performance and reported concerns. This example illustrates a possible clinical application; such patterns were not examined in the present study. The analyses also did not establish whether combined assessment improves clinical outcomes or fall prediction, or whether strength, mobility, or balance interventions reduce FOF.

Several limitations should be acknowledged. First, the cross-sectional design precludes causal and temporal inferences. Second, all regression models were unadjusted. Although falls during the previous 12 months were recorded, fall history was used descriptively; confounding by prior falls, age, sex, BMI, and other factors cannot be excluded. Third, the sample comprised 73 participants from four centers in one region and included predominantly women. Eligibility requirements for independent walking and an MMSE-K score of ≥20 further limit generalizability to other care populations. Fourth, self-reported FOF and retrospective fall history may be affected by response and recall bias, respectively. Finally, the strength and balance assessments captured selected aspects of physical function and did not provide a comprehensive evaluation of lower limb function or postural control. Larger longitudinal studies with prespecified covariate-adjusted analyses are needed to examine whether these associations persist and to clarify their temporal relationships.

## 5. Conclusions

Among older adults attending community-based day-care centers, lower KES, longer TUG completion times, and shorter FRT distances were associated with higher FES-I scores in unadjusted analyses. These findings support the potential relevance of jointly assessing strength, mobility, balance control, and FOF in this population. Further longitudinal studies with prespecified covariate-adjusted analyses are needed to determine whether these associations persist after adjustment and to clarify their temporal relationships.

## Figures and Tables

**Table 1:** General characteristics of participants (*n* = 73).

Variable	Mean ± SD/*n* (%)
Age (years)	79.2 ± 5.1
Sex (male/female)	17 (23.3)/56 (76.7)
Height (cm)	155.6 ± 6.7
Weight (kg)	56.4 ± 8.9
BMI (kg/m^2^)	23.4 ± 3.2
KES (kgf)	11.8 ± 4.3
TUG (s)	13.6 ± 3.8
FRT (cm)	20.9 ± 6.4
FES-I (score)	33.5 ± 9.1
Fall experience (yes)	25 (34.2)

BMI, body mass index; KES, knee extension strength; TUG, Timed Up and Go; FRT, Functional Reach Test; FES-I, Falls Efficacy Scale-International.

**Table 2:** Correlation between lower limb strength, mobility, balance, and fear of falling (*n* = 73).

Variable	KES	TUG	FRT	FES-I
KES	1	−0.52 **	0.48 **	−0.42 **
TUG		1	−0.45 **	0.39 **
FRT			1	−0.36 **
FES-I				1

** *p* < 0.01.

**Table 3:** Simple linear regression analyses of physical factors associated with fear of falling (FES-I) (*n* = 73).

Independent Variable	β	t	*p*	R^2^
KES (kgf)	−0.421	−3.91	<0.001	0.177
TUG (s)	0.394	3.61	<0.001	0.155
FRT (cm)	−0.361	−3.26	0.002	0.130

KES, knee extension strength; TUG, Timed Up and Go; FRT, Functional Reach Test; FES-I, Falls Efficacy Scale-International; β, standardized regression coefficient; R^2^, coefficient of determination. All models were unadjusted; *n* = 73.

## Data Availability

The datasets presented in this article are not readily available because of privacy and ethical restrictions related to the study participants. Requests to access the datasets should be directed to the corresponding author.

## Abbreviations

The following abbreviations are used in this manuscript:

Abbreviation	Full Term
FOF	Fear of Falling
KES	Knee Extension Strength
TUG	Timed Up and Go
FRT	Functional Reach Test
FES-I	Falls Efficacy Scale-International
KFES-I	Korean Version of the Falls Efficacy Scale-International
MMSE-K	Korean Version of the Mini-Mental State Examination
BMI	Body Mass Index
IMU	Inertial Measurement Unit
AI	Artificial Intelligence
STROBE	Strengthening the Reporting of Observational Studies in Epidemiology
IRB	Institutional Review Board
SD	Standard Deviation
